# Evaluation of Anticancer Activity of 76 Plant Species Collected in Andalusia (Spain) against Lung Cancer Cells

**DOI:** 10.3390/plants12183275

**Published:** 2023-09-15

**Authors:** Víctor Jiménez-González, Guillermo Benítez, Julio Enrique Pastor, Miguel López-Lázaro, José Manuel Calderón-Montaño

**Affiliations:** 1 Department of Pharmacology, Faculty of Pharmacy, University of Seville, 41012 Seville, Spain; mlopezlazaro@us.es; 2 Department of Botany, Faculty of Pharmacy, University of Granada, 18071 Granada, Spain; gbcruz@ugr.es; 3 Department of Vegetal Biology and Ecology, Faculty of Biology, University of Seville, 41012 Seville, Spain; jpastor@us.es

**Keywords:** cancer, lung cancer, selectivity, *Xiphion xiphium*, *Iris xiphium*, Iridaceae

## Abstract

Every year, cancer kills millions of people around the world. Finding more selective anticancer agents is essential to improve the low survival rates of patients with metastatic cancers. Since the research of natural products is a valuable approach to the discovery of new compounds and the Iberian flora offers a rich source of unstudied plants, we have carried out a random screening of 76 plant species from 43 families collected in Andalusia (South of Spain). Using non-malignant cells (HaCaT) and lung cancer cells (A549), we found that the extract from *Arum italicum* Mill. subsp. *italicum* (Araceae), *Mandragora autumnalis* Bertol. (Solanaceae), *Rhamnus alaternus* L. (Rhamnaceae), and *Lomelosia simplex* (Desf.) Raf. subsp. *dentata* (Jord. & Fourr.) Greuter & Burdet (Dipsacaceae) showed selective cytotoxicity against lung cancer cells. Extracts of plant species belonging to the Iridaceae family showed high selective activity against cancer cells, highlighting that the *Xiphion xiphium* (L.) M.B. Crespo, Mart.-Azorín & Mavrodiev flower extract was more selective against lung cancer cells than the standard anticancer drugs, cisplatin and 5-fluorouracil. This extract also showed modest selective cytotoxicity against bladder carcinoma cells (T24). The number of cells in the G1 phase increased after treatment with the extract from *Xiphion xiphium*. Our research indicates that various plants are potential sources for the isolation and development of new anticancer drugs.

## 1. Introduction

Cancer is a major public issue worldwide and is the second leading cause of death behind cardiovascular disease in many countries [1,2]. Due to significant improvement in the prevention and treatment of cardiovascular diseases and the increasing aging of the population, cancer will become the first leading cause of death in the next few years. According to estimates from the World Health Organization, there were 19.3 million new cases and 10 million cancer deaths in 2020. Although many tumors can often be cured if treated when the cancer cells are still located in the original tissue, most patients present metastasis when cancer is diagnosed [3]. At this stage of the disease, cancer cells have spread from their site of origin to another part of the body, requiring treatment with systemic drugs to reach them. Traditional or standard chemotherapy uses drugs that are cytotoxic and kill highly proliferative cells, such as cancer cells. However, these drugs also damage normal cells, causing numerous adverse effects that force the use of tolerated doses, which are usually insufficient to eradicate cancer cells. Recent advances in understanding the biology of the disease and the immune escape mechanisms of cancer cells have led to the development of targeted therapies (such as tyrosine kinase inhibitors) and immunotherapy (such as immune checkpoint inhibitors) that have overcome the efficacy of standard chemotherapy [4,5,6,7]. These new drugs are better tolerated and increase the survival of patients and their quality of life. However, they are only applicable to types of cancers with specific genes or proteins, and most metastatic cancers are still uncurable. In fact, patients diagnosed with the most common metastatic cancers have very low 5-year survival rates [1,2]. For example, the survival rates for patients with distant metastases are 32% in melanoma, 32% in prostate cancer, 30% in breast cancer, 15% in renal cancer, 14% in colorectal cancer, 7% in lung cancer, and 3% in liver cancer [2]. In addition to the limited efficacy of existing therapies, it is also important to mention that these therapies are very expensive and not all patients can afford these treatments [6]. For all these reasons, it is important to find new anticancer drugs with high selectivity against cancer cells to develop more effective and cheaper treatments for patients with metastatic cancer [8,9,10].

The Plant Kingdom has been a source of many anticancer drugs clinically approved, both natural and semisynthetic, over the years [11,12]. Some of these anticancer agents are considered essential medicines by the World Health Organization [13]. For example, vincristine and vinblastine, isolated from *Catharanthus roseus* (L.) G. Don [14,15], are useful agents against various types of lymphomas and solid tumors. The semisynthetic derivative of vinca alkaloids, vinorelbine, was synthesized by Pierre Potier and his team in 1989, and it is a very useful clinical antimitotic drug used to treat non-small cell lung cancer (NSCLC) [12,16]. The topoisomerase inhibitor etoposide, a semisynthetic analogue of podophyllotoxin from *Podophyllum peltatum* L., is approved for the treatment of several types of cancer, including NSCLS [17]. It is also important to mention that some of these natural compounds were identified via a systematic screening of plant extracts, such as camptothecin (a topoisomerase I inhibitor from the bark of *Camptotheca acuminata* Decne.) [18] and paclitaxel (an antimitotic drug isolated from *Taxus brevifolia* Nutt.) [19]. Although camptothecin is not used clinically, its analogues, irinotecan and topotecan, are widely used to treat cancer patients [18,20,21]. Paclitaxel and its semisynthetic derivative, docetaxel [22], are widely used antimitotics in the treatment of cancer, for example, in NSCLC [23]. 

Although several plants have provided useful anticancer agents, nature is being replaced by the new modern techniques for drug discovery (such as molecular docking, structure-based screening, homology modelling, etc. [24]). However, we cannot forget that less than 20% of plant species have ever been studied for potential therapeutic effects [25,26], and therefore, new natural anticancer potential compounds are still waiting to be discovered. 

Evidence suggests that the random screening of plants from areas with high levels of biodiversity and endemism may lead to novel drug discovery because the biodiversity of organisms can generate a variety of natural compounds, resulting in a broad spectrum of pharmacological activities. Andalusia, a southern region of the Iberian Peninsula, is home to a rich diversity of plants: 4437 taxa distributed in 171 families, 1107 genera, and 4091 species and 346 subspecies, of which 3958 are native and 479 alien [27,28,29]. Many of these plants have never been evaluated for their possible anticancer activity. We have previously reported the results of the evaluation of the selective anticancer activity of plants collected in several areas of Andalusia, reporting that plant species, such as *Daphne laureola* L., are potential sources of natural compounds with selective toxicity towards cancer cells [30,31]. In this work, we have continued our screening and evaluated the selective anticancer activity of 82 extracts from 76 plant species collected in several regions of Andalusia. The plants belong to 43 families. We evaluated the anticancer activity of these plant extracts in human lung cancer cells. We selected this type of cancer for our screening because it is the second most common cancer worldwide, being the leading cause of cancer-related deaths [1,2]. The reason for lung cancer lethality is due to the fact that over 65% of lung cancers are diagnosed in the advanced stages when treatment options are limited. Chemotherapy is the most common treatment option in these stages, highlighting that anticancer drugs most commonly used for lung cancer include drugs of natural origin or are derived from them (such as paclitaxel, docetaxel, etoposide, and vinorelbine). However, the current drugs do not usually cure the disease. As mentioned above, the five-year relative survival for patients with distant metastases is 7% in lung cancer. Therefore, new anticancer drugs are urgently needed for patients with lung cancer. 

## 2. Results and Discussion

Since the random screening of plants had led to anticancer drug discovery in the past, we have evaluated the selective anticancer activity of 76 plants growing in Andalusia, Spain. After collecting the plant material and preparing 82 extracts, we used human lung cancer cells (A549) and human non-malignant keratinocytes (HaCaT) to evaluate their selective cytotoxicity with the resazurin assay. Lung cancer is the leading cause of cancer deaths worldwide and NSCLC accounts for approximately 80–85% of all lung cancers [32]. A549 is one of the most widely used human NSCLC cell lines to evaluate anticancer drugs. To study selectivity, we used human keratinocytes HaCaT [33,34], a cell line derived from normal adult tissue that has a proliferation rate similar to that of cancer cells. Most anticancer drugs are not sufficiently selective, targeting both cancer cells and normal cells that have similar division rates. 

**Figure 1:**
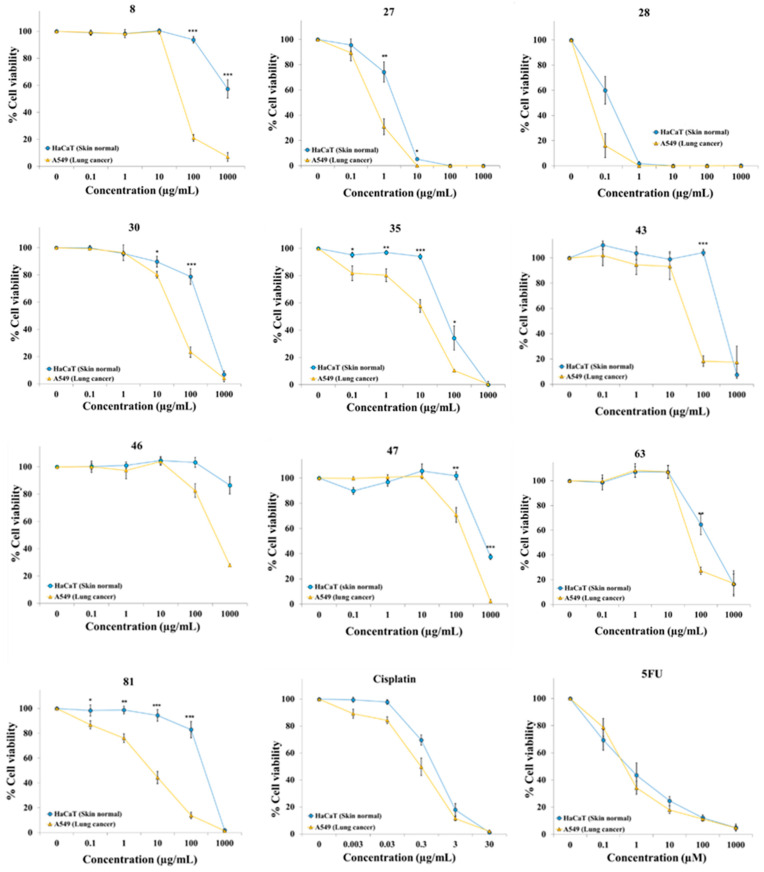
Cytotoxic activity of plant extracts **8**, **27**, **28**, **30**, **35**, **43**, **46**, **47**, **63**, and **81**, and anticancer standard drugs (cisplatin and 5FU) on A549 lung cancer cells and HaCaT non-malignant cells. Cells were exposed for 72 h to extracts and cell viability was determined with the resazurin assay. Data represent mean ± SEM from at least three independent experiments. Statistical analysis was calculated using the paired *t*-test; * indicates *p* < 0.05, ** indicates *p* < 0.01, *** indicates *p* < 0.001.

Both cell lines were treated with different concentrations of the extracts for 72 h before quantifying cell viability. Cisplatin and 5-fluorouracil (5FU), two well-known standard anticancer drugs, were used as positive controls. Table 1 displays the scientific names of the plants in alphabetical order, plant families, part of the plant used to elaborate the extract, voucher number, origin, IC_50_ values for each cell line, and selectivity indices. The concentration–response curves for the 82 extracts and positive controls are shown in Figure 1 and Figures S1–S7. These graphs allow for the easy visualization and understanding of the cytotoxic profile over a wide range of concentrations.

These results show that several extracts induced selective cytotoxicity against the lung cancer cell line A549 (Figure 1). Extract **46** and **47** from *Mandragora autumnalis* Bertol. (Solanaceae), extract **63** from the leaves of *Rhamnus alaternus* L. (Rhamnaceae), and extract **43** from aerial part of *Lomelosia simplex* (Desf.) Raf. subsp. *dentata* (Jord. & Fourr.) Greuter & Burdet (Dipsacaceae) were 2.7-, 3.5-, 4.2-, and 8.2-fold more cytotoxic against cancer cells than to non-malignant cells, respectively. Extract **8** from the aerial parts of *Arum italicum* Mill. subsp. *italicum* (Araceae) was over 20 times more active against A549 lung cancer than against HaCaT non-malignant cells. The extracts from plant species belonging to the Iridaceae family (**27**, **28**, **30**, **35**, and **81**) showed high selective activity against the cancer cells. Extract **27** from whole plant *Gynandriris sisyrinchium* (L.) Parl. was approximately four times more selective against lung cancer cells A549. Extract **30** from *Iris germanica* L. and extract **35** from *Juno planifolia* (Mill.) Asch. were at least 7-fold more active against lung cancer cells. Interestingly, extract **81** from the flowers from *Xiphion xiphium* (syn. *Iris xiphium* L.) was more selective against lung cancer cells than standard anticancer drugs cisplatin and 5FU. A549 cancer cells were almost 40 times more sensitive than HaCaT cells, with IC_50_ values (mean ± SEM; μg/mL) 8.6 ± 2.1 and 250.2 ± 29.5, respectively. Many extracts were cytotoxic but not selective against cancer cells, for example, in extracts **9**, **10**, **14**, **19**, **20**, **21**, **22**, **23**, **25**, **33**, **34**, **37**, **49**, **73**, **74**, **77**, **78**, and **79** and even some others (**1**, **11, 13, 15, 18**, **45**, and **80**) were clearly more toxic against normal cells than against cancer cells (Figures S1–S7). Several extracts (**6**, **16**, and **48**) did not show clear cytotoxicity at the maximum concentration tested (1000 μg/mL) for any of the cell lines (Figures S1–S7). 

**Figure 2:**
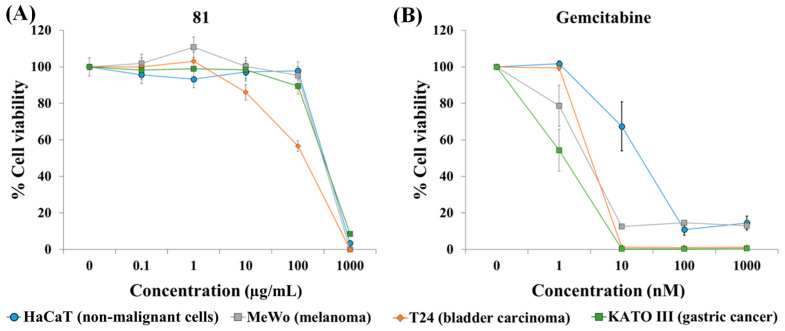
Evaluation of the selective anticancer activity of extract **81** (**A**) and gemcitabine (**B**) on human non-malignant cells (HaCaT) and human cancer cells (MeWo, T24 and KATO III). Cells were exposed to several concentrations of extract and gemcitabine for 72 h. After treatment, cell viability was determined with the resazurin assay. Data represent mean ± SEM from at least three independent experiments.

Due to the fact that the extract from the flowers of *Xiphion xiphium* (**81**) was the most selective anticancer extract, we decided to test whether this extract was also active against other types of cancer. MeWo melanoma cells, T24 bladder carcinoma cells, KATO III gastric cancer cells, and HaCaT non-malignant cells were exposed to several concentrations of extract **81** for 72 h. After treatment, cell viability was estimated with the resazurin assay (Figure 2 and Table 2). Extract **81** showed modest selectivity against T24 bladder carcinoma cells, being approximately 2.6 times more cytotoxic against this cancer cell line than against HaCaT cells. However, this extract was not selective against MeWo melanoma cells and KATO III gastric cancer cells. The anticancer drug gemcitabine showed selective anticancer activity against the three cancer cell lines. We also tested extract **81** on BJ-hTERT cells (hTERT-immortalized foreskin fibroblast BJ cells). These cells are derived from normal foreskin BJ cells transformed genetically using hTERT (human telomerase reverse transcriptase) to avoid senescence, and they are non-tumorigenic. After a 72 h treatment, the IC_50_ value was 201.0 ± 46.6 µg/mL (Figure S8). These results suggest that plant extract **81** is more active against lung cancer than other types of cancer. For this reason, we delved into its anticancer activity using A549 lung cancer cells. 

**Figure 3:**
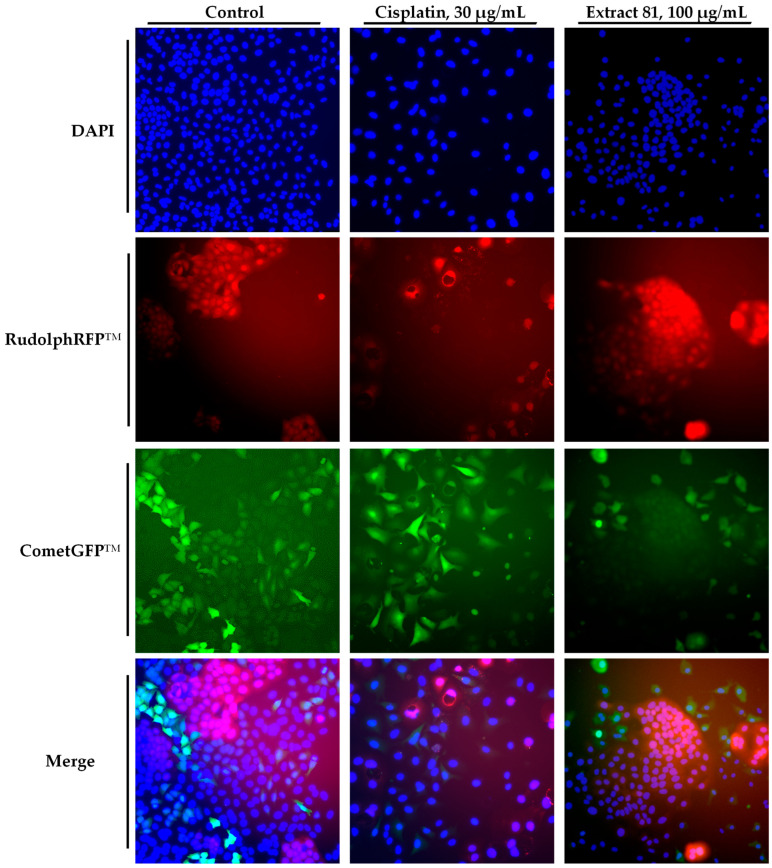
Representative photographs of untreated cells (control), cells treated with the positive control cisplatin, and cells exposed to extract **81** for 72 h. Cell viability determined via the resazurin assay. Images were taken using a Nikon Eclipse Ti-E epifluorescense microscope (magnification 20×). HaCaT-GFP-RFP non-malignant cells show dual-labelling (red and green fluorescence) due to expression of RudolphRFP and CometGFP, and A549-GFP lung cancer cells appear as green fluorescence due to expression of CometGFP.

**Figure 4:**
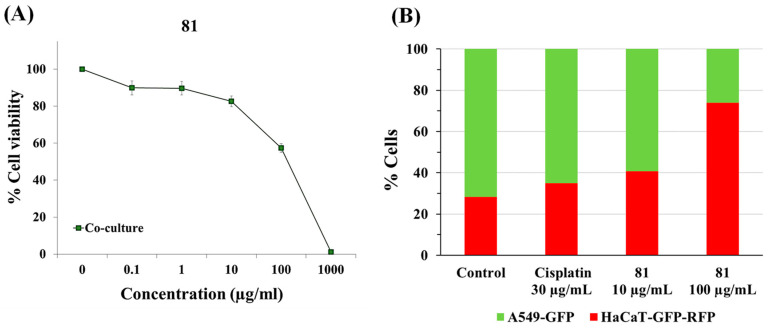
Cytotoxic activity of extract **81** from the flowers of *Xiphion xiphium* and cisplatin on co-culture of A549-GFP and HaCaT-GFP-RFP cells. Cells were treated with cisplatin or extract **81** for 72 h. (**A**) Cell viability determined via the resazurin assay. (**B**) Quantification of percentage of cells. Data represent mean ± SEM from at least two independent experiments.

Next, we wanted to confirm the selective anticancer activity of plant extract **81** via the co-culture of transformed GFP-overexpressing cancer cells (A549-GFP) and GFP-RFP-overexpressing non-malignant cells (HaCaT-GFP-RFP). Both cell lines were co-cultured and exposed to several concentrations of extract **81** and cisplatin for 72 h. After treatment, cell viability was determined with the resazurin assay. The cells were then washed and fixed with cold ethanol. After fixation, DNA was stained with DAPI and the cells were observed at 20-fold magnification with a fluorescence microscope. Figure 3 shows representative photographs of the co-culture exposed to extract **81** or cisplatin and Figure 4 shows the quantification of the number of cells for each type of cell line. We observed that A549-GFP cancer cells grew faster and were the majority population of cells in untreated samples (71.6% A549-GFP versus 28.4% HaCaT-GFP-RFP). Interestingly, after treatment with extract **81**, the population of A549-GFP cells decreased in a dose-dependent manner, without significantly affecting the population of HaCaT-GFP-RFP. The co-culture treated with 100 µg/mL of extract **81** showed approximately 60% cell viability compared to the untreated co-culture (Figure 4A) being 74% HaCaT-GFP-RFP and 26% A549-GFP (Figure 4B). Cisplatin similarly affected both populations of cells. 

**Figure 5:**
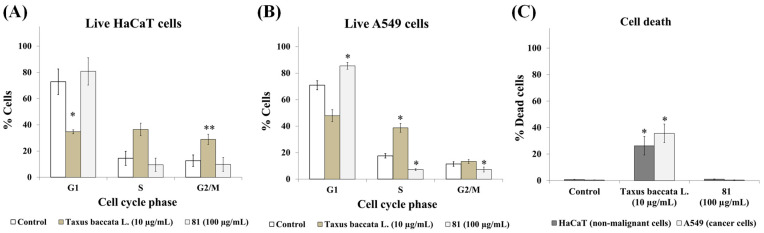
Treatment with extract **81** (*Xiphion xiphium*) results in G1 phase arrest in A549 cells. HaCaT cells and A549 cells were treated with the indicated extract for 72 h and propidium iodide staining was carried out to measure cell-cycle profile using flow cytometry. (**A**,**B**) Cell cycle analysis of the live cells. (**C**) The percentage of dead cells was calculated based on the number of cells with less than 2N (SubG1) DNA content. Data represent mean ± SEM from at least three independent experiments. Statistical analysis was calculated using the paired *t*-test; * indicates *p* < 0.05, ** indicates *p* < 0.01, versus control.

These results indicate that extract **81** has selective activity against lung cancer cells. We next investigated the effects of this extract on the cell cycle in HaCaT and A549 cells (Figure 5). The extract from the leaves of *Taxus baccata* L. (Taxaceae) was used as a positive control. We previously showed that this extract has selective activity against A549 cancer cells [31], and it is known that *Taxus baccata* contains different taxane-type diterpenes, including paclitaxel [35]. This extract induced severe S and G2/M phase arrest and death cell, as was expected by its content in antimitotic compounds. While treatment with extract **81** (100 µg/mL) for 72 h had very little effect on the cell-cycle profile of HaCaT non-malignant cells, this extract induced a statistically significant G1 phase arrest in A549 cancer cells. Treatment with this extract did not increase the number of cells in the sub-G1 (apoptotic) phase in any cell line. These data suggest that the effect of extract **81** may be cytostatic instead of cytotoxic against A549 cells. However, this effect may depend on the degree of malignancy of the cancer cell line. Shin et al. [36] observed that an ethanol extract of *Xiphion orientale* Schrank (syn. *Iris nertschinskia* G. Lodd.) induced G1 phase arrest in breast cancer MDA-MB-231 and Hs578T cells, but the treatment with this extract increased the number of cells in the subG1 (apoptotic) in MDA-MB-231 cells, but not in Hs578T.

In this work, we show that several extracts from the Iridaceae family belonging to Iris and its related genus (**27**, **28**, **30**, **35**, and **81**) induced selective cytotoxicity to human lung cancer cells (A549). Recently, the Iris genus has been split into more than 20 different genera using molecular techniques, constituting a polyphyletic group instead of a monophyletic [37]. Due to the lack of information available on phytochemistry and pharmacology about the new genera, we focus our research on understanding the activity described in this article within the traditional concept and scientific knowledge of Iris as a monophyletic genus. Other species of Iris and its related genus have previously been studied by other authors [36,38,39,40,41,42,43]. For example, extracts from the rhizome of the Iris species (*I. crocea* Jacquem. ex R.C.Foster, *I. ensata* Thunb [*Xiphion donianum* Alef.], *I. germanica* L., *I. hungarica* Waldst. & Kit., *I. kashmiriana* Baker, *I. nertchinskia* G.Lodd. [*Xiphion orientale* Schrank], *I. pseudopumila* Tineo, *I. spuria* L. [*Xiphion spurium* Alef.], and *I. variegata* L) have shown cytotoxic activity against various cancer cell lines, such as breast cancer (MCF7, MDA-MB-231), colon cancer (Caco-2, HCT116), cervical adenocarcinoma (Hela), melanoma (C32, IGR39), renal carcinoma (ACHN), and lung cancer (A549, CORL-23) [38,39,40,41]. One of the most famous plants of the Iridaceae family is *Crocus sativus* L. Saffron, the most expensive spice, which is obtained from the dried stigma of this plant and exhibits several pharmacological effects, including anticancer activity [44]. Several types of phytochemicals can be involved in the anticancer activity shown by these plants, for example, some phenolic acids (gallic acid and sinapic acid) [45,46,47], flavone (embigenin, luteolin, and embinin) [48,49,50,51], isoflavones (irishkumaonin methyl ether, iristectorin B, tectorigenin, daidzein, and iriflogenin) [45,52,53,54], flavonols (irisoid A, quercetin, myricetin, kaempferol, (+)-catechin, and (−)-epicatechin) [47,51,54,55,56], xanthones (mangiferin and isomangiferin) [45,54], and triterpenoids known as iridals (iridal, iriflorental, α-irigermanal, γ-irigermanal, and 16-acetoxyiridal). Some of these compounds, alone or in combination with other compounds, could be responsible for the selective activity of extract **81** (*X. xiphium*). It should be mentioned that extract **81** showed a much lower value of IC_50_ (8.6 µg/mL) against cancer cells than extracts from rhizomes or whole plants from other species of Iris [50,51], suggesting the potential anticancer of this plant. 

## 3. Materials and Methods

### 3.1. Plant Material

All plants were collected by V. Jiménez-González between July 2018 and August 2021 in Huelva and Sevilla (Andalusia, Spain). Plant samples (5–70 g) were carefully obtained to avoid any damage that could affect the conservation of any species. Voucher specimens were deposited in the herbarium of the University of Seville (SEV according to [57], located at CITIUS II Celestino Mutis, Centre for Research, Technology, and Innovation). The scientific nomenclature of the wild plants has been updated to the latest publication on the matter by Cueto et al. [29]. Scientific names, selected plant parts, and voucher numbers are displayed in Table 1. Collection coordinates are shown in Table S1.

### 3.2. Preparation of the Extracts

Plant extracts were made within several hours after the collection of the plant material. From 100 to 200 mL of a mixture of ethanol/ethyl acetate/water (1:1:1) was added to the fresh plant material (5–70 g) to start the extraction at 60 °C for 1 h in an ultrasound water bath apparatus. After vacuum filtration, the ethyl acetate and ethanol solvents were eliminated in a rotary vacuum evaporator at 60 °C. Finally, the dried extracts were obtained via lyophilization. The extraction yield (%) for each extract is shown in Table S2. 

The extracts were stored in amber glass bottles and kept in a cool dark place. The first cytotoxicity assay was performed in the next month after preparation to avoid any degradation of the compounds. For the cytotoxicity assay, each dry extract was dissolved with DMSO to prepare a stock solution (100 mg/mL). The working solutions of specific concentrations were prepared via the dilution of the stock solutions in the culture medium and were immediately used to treat the cells. The remaining stock solutions were aliquoted and frozen at −80 °C. We used different aliquots in each independent cytotoxicity experiment to avoid the freeze—thaw cycles. 

### 3.3. Drugs and Reagents 

Cisplatin and 4′,6′-Diamidino-2-phenylindole dihydrochloride (DAPI, 202710100) were bought from Thermo Scientific Acros Organics (Waltham, MA, USA). Gemcitabine was obtained from Pfizer S.L. (Madrid, Spain). Rezasurin (R7017), ECOSURF™ EH-9 (A9778), and Ribonuclease A (R4642) were purchased from Sigma Aldrich (St. Louis, MO, USA). JetPEI poly transfection reagent (101-10N) was acquired from Polyplus (Illkirch, France). PB-GFP plasmid (pHULK piggyBac Mammalian Expression Vector–CometGFP™, pJ503-02) and PB-RFP plasmid (pHULK piggyBac Mammalian Expression Vector–RudolphRFP™-IRES-CometGFP™, pJ549-17) were purchased from DNA2.0 (Menlo Park, CA, USA). Corning™ G418 Sulfate (30-234-CR) and Puromycin (J67236.XF) were purchased from Thermo Scientific (Waltham, MA, USA). Propidium iodide (A2261) was bought from Panreac Applichem (Darmstadt, Germany).

### 3.4. Cell Lines

HaCaT (non-malignant human keratinocytes, 300493 [58]), A549 (human non-small-cell lung cancer cells, 300114), T24 (human bladder cancer cells, 300352), KATO III (human gastric cancer cells, 300381), and MeWo (human melanoma cells, 300285) were purchased from the Cell Line Services (CLS, Hamburg, Germany). BJ-hTERT cells were kindly provided by Dr. Hahn (Dana-Farber Cancer Institute, Boston, MA, USA).

A549-GFP and HaCaT-GFP-RFP were generated via stable transfection with PB-GFP or PB-RFP plasmids, respectively. These plasmids were transfected into cells using the JetPEI poly transfection reagent. 300,000 cells per well were seeded in 6-well plates. When the cells were 50% confluent, they were transfected with 2 µg of DNA and 4 µL of JetPEI for 4 h. Then, the medium was changed and the cells were expanded for 8 days before their selection. Puromycin (0.5 µg/mL) and Corning™ G418 Sulfate (250 µg/mL) were used to select HaCaT-GFP-RFP and A549-GFP, respectively. 

The cells were maintained in Dulbecco’s modified Eagle medium (DMEM) containing 4.5 g/L of D-glucose and L-glutamine, but no sodium pyruvate. All media were supplemented with penicillin (100 U/mL), streptomycin (100 µg/mL), and fetal bovine serum (10%). The cells were cultured in a humidified CO_2_ atmosphere at 37 °C. Cell culture reagents were obtained from Biowest (Nuaillé, France) and Thermo Fisher Scientific, unless otherwise indicated. 

### 3.5. Cell Viability Assay

The resazurin assay is widely used to measure cell viability. This assay is based on the ability of viable cells to convert the blue compound resazurin into a pink, fluorescent, and soluble compound resorufin. The amount of resorufin produced is generally proportional to the number of viable cells. This assay was carried out as previously described [59,60]. Briefly, exponentially growing cells (3000–5000 cells per well) were seeded in 96-well plates. At 24 h after seeding, the cells were exposed to several concentrations of plant extracts or anticancer drugs (cisplatin, 5FU, and gemcitabine). After a 72 h treatment period, the medium was removed, and the cells were washed once with a phosphate-buffered saline (PBS). Then 150 μL of resazurin (20 μg/mL in the medium) was added to each well. Since KATO III cells grow as a mixture of suspension and adherent cells, 50 μL of resazurin (60 μg/mL in the medium) was added directly without prior washing. The plates were incubated for 4–5 h (depending on the cell line) at 37 °C, 5% CO_2_, and, finally, the optical densities were measured at 540 and 620 nm using a multiwell plate spectrophotometer reader (Imark Bio Rad Laboratories Inc., Hercules, CA, USA).

Cell viability was obtained as a percentage related to untreated cells. The results were expressed as mean ± standard error of the mean (SEM) of at least two independent experiments. The selectivity indices (S.I.) were used to analyze the selective anticancer activity of plant extracts. The S.I. was calculated for each cancer cell line as the average of the IC_50_ value in the non-malignant cell line (HaCaT) divided by the IC_50_ value in the respective cancer cell line obtained in each independent experiment.

### 3.6. Co-Culture Assay

The A549-GFP and HaCaT-GFP-RFP cells were co-cultured in 96-well plates at a density of 3000 cells per well for each cell line. The cells were allowed to grow for 24 h and were then exposed to different concentrations of cisplatin and extract **81** (*X. xiphium*). After 72 h of treatment, the cells were fixated with 70% ethanol, DNA was stained with DAPI, and the cells were observed at a 20-fold magnification with a Nikon Eclipse Ti-E epifluorescense microscope. A total of 150–300 cells/well were counted. 

### 3.7. Flow Cytometry

The cells (200,000 cells per well) were seeded in six-well plates and kept for overnight incubation. The next day, the medium was removed and replaced with a fresh medium. The cells were treated with the *X. xiphium* (**81**) extract or the positive control *Taxus baccata* extract. After 72 h of treatment, the supernatant as well as the trypsinized single-cell suspension were collected. The samples were spun down (300 G for 4 min at 4 °C) and washed twice with cold PBS. The cells were fixed in 70% cold ethanol and kept at 4 °C for 60 min. After washing with PBS, the cells were resuspended in PBS containing 30 µg/mL of propidium iodide, 0.1% Tween 20, 0.1% ECOSURF™ EH-9, and 10 µg/mL of Ribonuclease A for 60 min at 4 °C. The cell cycle profiles were analyzed using a Beckman Coulter CYTOMICS FC500 (High Wycombe, UK).

### 3.8. Statistical Analysis

The *t*-test (paired and two tailed) was used for statistical analysis. A *p* value > 0.05 is not considered statistically significant and is not represented by any symbol. A *p*-value ≤ 0.05 was considered statistically significant and was shown with an asterisk, two asterisks (*p* ≤ 0.01), or three asterisks (*p* ≤ 0.001). 

## 4. Conclusions

Since the random screening of plants had led to anticancer drug discovery in the past, we have studied the anticancer activity of a variety of plants collected in the south of Spain. After collecting 76 plants from 43 families and preparing 82 extracts, we evaluated their potential anticancer effect using the lung cancer cell line A549 and the non-malignant cell line HaCaT. Several extracts showed selective toxicity against cancer cells, including extracts of *Arum italicum* subsp. *italicum*, *Gynandriris sisyrinchium*, *Iris germanica*, *Lomelosia simplex* subsp. *dentata*, *Juno planifolia*, *Mandragora autumnalis*, *Rhamnus alaternus*, and *Xiphion xiphium*. Since the extract of *Xiphion xiphium* was the most selective against lung cancer cells, we evaluated it in an additional three cancer cell lines also showing a cytotoxic effect against T24 urinary bladder cancer cells. Our research indicates that various plants have potential as sources for the isolation and development of new anticancer drugs with selective toxicity toward cancer cells. More studies are needed to understand the nature and complexity of the compounds behind their potential anticancer activity and their mechanisms of action for the treatment of cancer.

## Figures and Tables

**Table 1:** Cytotoxic activity of plant extracts on lung cancer cells (A549) versus non-malignant cells (HaCaT).

Extract	Plant Name	Family	Part Used	Voucher Number (SEV)	Origin	IC_50_ (Resazurin) (Mean ± SEM, µg/mL)		Selectivity Index (Mean ± SEM)
						A549 (Cancer)	HaCaT (Non-Malignant)	
**1**	*Acoelorraphe wrightii* (Griseb. & H.Wendl.) H.Wendl. ex Becc. *	*Arecaceae*	Leaf	289245	Sevilla	352.8 ± 44.1	41.6 ± 5.3	0.1 ± 0.0
**2**	*Aegilops geniculata* Roth	*Poaceae*	Whole plant	289701	Sevilla	272.5 ± 25.3	338.6 ± 18.4	1.6 ± 0.8
**3**	*Alkanna tinctoria* (L.) Tausch	*Boraginaceae*	Aerial part with flowers	289283	Sevilla	308.3 ± 121.7	220.8 ± 13.7	1.0 ± 0.3
**4**	*Alyssum simplex* Rudolphi	*Brassicaceae*	Whole plant	289284	Sevilla	>1000	>1000	N.D.
**5**	*Amaryllis belladonna* L. *	*Amaryllidaceae*	Root	289734	Sevilla	8.5 ± 2.0	20.4 ± 3.5	3.3 ± 0.9
**6**	*Arisarum simorrhinum* Durieu	*Araceae*	Aerial parts with flowers	288075	Sevilla	>1000	>1000	N.D.
**7**	*Aristolochia paucinervis* Pomel	*Aristolochiaceae*	Aerial part with flowers	289282	Sevilla	19.1 ± 5.1	21.7 ± 2.1	1.5 ± 0.5
**8**	*Arum italicum* Mill. *subsp. italicum*	*Araceae*	Aerial parts	288072	Sevilla	43.8 ± 2.7	>1000	>22.8
**9**	*Bartsia trixago* L.	*Orobanchaceae*	Aerial part with flowers	289294	Sevilla	353.6 ± 16.7	380.6 ± 17.9	0.9 ± 0.2
**10**	*Bolboschoenus maritimus* (L.) Palla	*Cyperaceae*	Aerial part with flowers	289287	Sevilla	247.1 ± 20.2	248.2 ± 16.0	1.0 ± 0.1
**11**	*Brachychiton populneus* R.Br. *	*Malvaceae*	Aerial parts with fruits	289251	Sevilla	270.8 ± 62.0	75.4 ± 14.1	0.5 ± 0.2
**12**	*Briza maxima* L.	*Poaceae*	Aerial part	289290	Sevilla	472.4 ± 98.0	350.8 ± 20.7	0.6 ± 0.2
**13**	*Butia capitata* (Mart.) Becc. *	*Arecaceae*	Leaf	289801	Sevilla	110.0 ± 40.1	37.1 ± 3.0	0.5 ± 0.2
**14**	*Catalpa bignoniodes* Walter *	*Bignoniaceae*	Leaf	289259	Sevilla	65.9 ± 25.6	96.4 ± 53.1	1.9 ± 1.2
**15**	*Ceiba speciosa* (A.St.-Hil.) Ravenna *	*Malvaceae*	Aerial part	289249	Sevilla	230.5 ± 61.0	38.2 ± 4.8	0.2 ± 0.1
**16**	*Celtis australis* L.	*Cannabaceae*	Fruits	289272	Sevilla	>1000	>1000	N.D.
**17**	*Celtis australis* L.	*Cannabaceae*	Aerial part	289272	Sevilla	394.8 ± 94.3	255.3 ± 55.2	0.7 ± 0.2
**18**	*Centranthus calcitrapae* (L.) Dufr.	*Valerianaceae*	Aerial part with flowers	289705	Sevilla	572.8 ± 118.1	210.9 ± 32.9	0.5 ± 0.1
**19**	*Cerinthe major* L.	*Boraginaceae*	Aerial parts with flowers	288089	Sevilla	170.8 ± 48.2	167.6 ± 52.4	1.5 ± 0.8
**20**	*Ceterach officinarum* Willd. subsp. *officinarum*	*Aspleniaceae*	Aerial parts	288074	Sevilla	179.1 ± 39.2	148.6 ± 42.4	1.4 ± 0.8
**21**	*Chamaerops humilis* L.	*Arecaceae*	Leaves	289731	Huelva	318.1 ± 20.8	538.1 ± 174.5	1.7 ± 0.5
**22**	*Chamaerops humilis* L.	*Arecaceae*	Fruits	289731	Huelva	235.7 ± 122.7	488.0 ± 264.1	1.7 ± 0.3
**23**	*Cuscuta campestris* Yunck.	*Cuscuteae*	Aerial part with flowers	289223	Sevilla	103.5 ± 43.6	181.5 ± 75.2	1.8 ± 0.5
**24**	*Dipcadi serotinum* (L.) Medik.	*Hyacinthaceae*	Whole plant	289805	Huelva	347.9 ± 34.9	261.4 ± 31.2	0.8 ± 0.1
**25**	*Fedia cornucopiae* (L.) Gaertn.	*Caprifoliaceae*	Whole plant	288077	Sevilla	388.5 ± 186.4	590.8 ± 487.9	1.0 ± 0.9
**26**	*Firmiana simplex* (L.) W.Wight *	*Malvaceae*	Leaf	289258	Sevilla	412.9 ± 72.3	379.1 ± 29.2	0.7 ± 0.3
**27**	*Gynandriris sisyrinchium* (L.) Parl.	*Iridaceae*	Whole plant	289804	Sevilla	0.6 ± 0.1	2.2 ± 0.4	4.5 ± 1.2
**28**	*Gynandriris sisyrinchium* (L.) Parl.	*Iridaceae*	Flowers	289804	Sevilla	˂ 0.1	0.2 ± 0.1	>2.8
**29**	*Heliotropium europaeum* L.	*Boraginaceae*	Aerial part with flowers	289273	Sevilla	257.2 ± 39.2	270.2 ± 41.8	1.3 ± 0.3
**30**	*Iris germanica* L.	*Iridaceae*	Root	289800	Sevilla	35.4 ± 3.9	255.2 ± 34.7	7.4 ± 1.1
**31**	*Jacaranda mimosifolia* D.Don *	*Bignoniaceae*	Flowers	289270	Sevilla	226.1 ± 41.0	338.8 ± 23.5	1.7 ± 0.4
**32**	*Jasminum fruticans* L.	*Oleaceae*	Fruits	288065	Sevilla	338.7 ± 1.7	471.9 ± 33.7	1.4 ± 0.1
**33**	*Jasminum fruticans* L.	*Oleaceae*	Aerial parts	288065	Sevilla	217.7 ± 42.3	262.7 ± 25.9	0.4 ± 0.1
**34**	*Juncus acutus* L. *subsp. acutus*	*Juncaceae*	Aerial part with fruits	289277	Sevilla	268.2 ± 92.2	228.8 ± 55.2	0.7 ± 0.2
**35**	*Juno planifolia* (Mill.) Asch.	*Iridaceae*	Aerial parts with flowers	289232	Sevilla	14.2 ± 2.7	76.5 ± 31.4	7.5 ± 3.5
**36**	*Koelreuteria paniculata* Laxm. *	*Sapindaceae*	Leaf	289246	Sevilla	30.4 ± 1.1	31.3 ± 2.0	1.0 ± 0.1
**37**	*Lagerstroemia indica* L. *	*Lythraceae*	Aerial part	289253	Sevilla	178.8 ± 71.1	205.0 ± 61.1	1.5 ± 0.9
**38**	*Lagerstroemia speciosa* (L.) Pers. *	*Lythraceae*	Leaf	289256	Sevilla	17.3 ± 2.7	25.1 ± 2.3	1.8 ± 0.5
**39**	*Lagunaria patersonia* (Andrews) G. Don *	*Malvaceae*	Leaf	289257	Sevilla	544.9 ± 143.1	475.2 ± 24.9	1.0 ± 0.2
**40**	*Linaria viscosa* (*L.*) *Chaz.*	*Veronicaceae*	Aerial parts with flowers	289231	Sevilla	151.8 ± 43.4	297.4 ± 11.6	2.8 ± 1.1
**41**	*Liquidambar styraciflua* L. *	*Altingiaceae*	Aerial part	289260	Sevilla	20.1 ± 1.9	23.5 ± 4.9	1.2 ± 0.3
**42**	*Lolium rigidum* Gaudin	*Poaceae*	Aerial parts	289697	Sevilla	>1000	>1000	N.D.
**43**	*Lomelosia simplex* (Desf.) Raf. subsp. *dentata* (Jord. & Fourr.) Greuter & Burde	*Dipsacaceae*	Aerial part with flowers	289708	Huelva	36.5 ± 2.6	375.8 ± 11.4	8.2 ± 2.9
**44**	*Lonicera implexa* Aiton	*Caprifoliaceae*	Leaves	288068	Sevilla	254.5 ± 17.9	164.2 ± 55.6	0.6 ± 0.2
**45**	*Maclura pomifera* (Raf.) C.K.Schneid. *	*Moraceae*	Aerial part	289252	Sevilla	496.9 ± 222.4	112.7 ± 56.3	0.4 ± 0.2
**46**	*Mandragora autumnalis* Bertol.	*Solanaceae*	Flower and fruits	288076	Sevilla	369.5 ± 42.1	>1000	>2.7
**47**	*Mandragora autumnalis* Bertol.	*Solanaceae*	Whole plant	288076	Sevilla	201.9 ± 30.7	645.7 ± 51.4	3.5 ± 0.7
**48**	*Morus nigra* L.	*Moraceae*	Fruits	289288	Sevilla	>1000	>1000	N.D.
**49**	*Muscari comosum* (L.) Mill.	*Hyacinthaceae*	Aerial parts with flowers	289234	Sevilla	331.4 ± 16.3	310.1 ± 4.6	0.9 ± 0.0
**50**	*Nonea vesicaria* (L.) Rchb.	*Boraginaceae*	Whole plant	288078	Sevilla	273.8 ± 36.9	315.5 ± 4.2	1.2 ± 0.2
**51**	*Oenothera rosea* L’Hér. ex Aiton *	*Onagraceae*	Aerial parts with flowers	289250	Sevilla	40.3 ± 19.1	29.6 ± 6.9	1.9 ± 1.4
**52**	*Ophrys scolopax* Cav.	*Orchidaceae*	Aerial part with flowers	289286	Sevilla	321.7 ± 25.4	303.5 ± 12.2	1.0 ± 0.1
**53**	*Ophrys speculum* Link	*Orchidaceae*	Whole plant	289281	Sevilla	415.1 ± 108.8	297.5 ± 20.4	0.8 ± 0.2
**54**	*Ornithogalum baeticum* Boiss. *	*Hyacinthaceae*	Whole plant	289280	Sevilla	22.0 ± 5.3	52.5 ± 11.8	3.3 ± 1.7
**55**	*Orobanche crenata* Forssk.	*Orobanchaceae*	Aerial parts with flowers	289235	Sevilla	328.6 ± 45.7	313.5 ± 36.8	1.0 ± 0.0
**56**	*Parentucellia viscosa* (L.) Caruel	*Orobanchaceae*	Aerial part with flowers	289289	Sevilla	414.6 ± 66.4	346.1 ± 14.0	0.7 ± 0.2
**57**	*Paronychia argentea* Lam.	*Caryophyllaceae*	Whole plant	289233	Sevilla	402.9 ± 70.8	263.4 ± 24.1	0.7 ± 0.1
**58**	*Petrorhagia nanteuilii* (Burnat) P.W.Ball & Heywood	*Caryophyllaceae*	Aerial part with flowers	289292	Sevilla	268.5 ± 11.5	337.8 ± 14.0	1.3 ± 0.1
**59**	*Photinia glabra* (Thunb.) Poit. *	*Rosaceae*	Aerial part	289254	Sevilla	233.6 ± 60.7	174.1 ± 35.4	0.9 ± 0.2
**60**	*Platanus hispanica* Mill. ex Münchh. *	*Platanaceae*	Leaf	289261	Sevilla	306.7 ± 45.4	315.9 ± 13.8	1.1 ± 0.1
**61**	*Platycapnos spicata* (L.) Bernh.	*Papaveraceae*	Aerial parts with flowers	288084	Sevilla	140.9 ± 47.6	308.5 ± 21.7	6.4 ± 4.5
**62**	*Plumbago europaea* L.	*Plumbaginaceae*	Aerial part with flowers and fruits	289271	Sevilla	1.8 ± 0.2	2.7 ± 0.2	1.6 ± 0.3
**63**	*Rhamnus alaternus* L.	*Rhamnaceae*	Leaves	289700	Sevilla	54.8 ± 3.5	234.2 ± 46.9	4.2 ± 0.8
**64**	*Rhamnus alaternus* L.	*Rhamnaceae*	Fruits	289700	Sevilla	490.7 ± 91.7	391.2 ± 32.5	0.6 ± 0.3
**65**	*Rosa canina* L.	*Rosaceae*	Fruits	289696	Sevilla	645.4 ± 59.7	>1000	>1.5
**66**	*Rumex conglomeratus* Murray	*Polygonaceae*	Aerial part with flowers	289710	Huelva	254.6 ± 69.1	207.0 ± 91.9	0.9 ± 0.2
**67**	*Schinus molle* L.	*Anacardiaceae*	Aerial part	289255	Sevilla	237.6 ± 69.9	216.5 ± 28.4	1.3 ± 0.3
**68**	*Scirpoides holoschoenus* (L.) Soják	*Cyperaceae*	Aerial part with fruits	289274	Sevilla	202.2 ± 54.8	204.1 ± 68.9	1.2 ± 0.6
**69**	*Scrophularia sambucifolia* L.	*Scrophulariaceae*	Aerial part with flowers	289285	Sevilla	360.7 ± 11.2	512.5 ± 104.6	1.4 ± 0.2
**70**	*Sedum amplexicaule* DC. subsp. *amplexicaule*	*Crassulaceae*	Whole plant	289293	Sevilla	298.2 ± 23.7	283.3 ± 32.2	1.0 ± 0.1
**71**	*Sedum mucizonia* (Ortega) Raym.-Hamet	*Crassulaceae*	Whole plant	289291	Sevilla	317.0 ± 16.8	332.5 ± 8.5	1.1 ± 0.1
**72**	*Solandra maxima* (Moc. & Sessé ex Dunal) P.S.Green *	*Solanaceae*	Leaves	288071	Sevilla	75.4 ± 15.9	154.2 ± 41.1	2.8 ± 1.1
**73**	*Solanum nigrum* L.	*Solanaceae*	Aerial parts	288073	Sevilla	36.7 ± 3.6	43.6 ± 7.6	1.1 ± 0.1
**74**	*Swietenia mahagoni* (L.) Jacq. *	*Meliaceae*	Leaf	289244	Sevilla	52.2 ± 9.4	47.0 ± 1.2	1.0 ± 0.2
**75**	*Syagrus romanzoffiana* (Cham.) Glassman *	*Arecaceae*	Aerial part	289802	Sevilla	226.5 ± 32.9	310.4 ± 10.1	1.5 ± 0.3
**76**	*Taxodium distichum* (L.) Rich. *	*Cupressaceae*	Aerial part	289264	Sevilla	301.2 ± 22.7	343.4 ± 8.1	1.2 ± 0.1
**77**	*Thymbra capitata* (L.) Cav.	*Lamiaceae*	Aerial part with flowers	289278	Sevilla	118.4 ± 34.7	99.4 ± 35.7	0.8 ± 0.1
**78**	*Tilia tomentosa* Moench. *	*Malvaceae*	Leaf	289265	Sevilla	292.6 ± 38.5	345.4 ± 13.7	1.4 ± 0.2
**79**	*Trachycarpus fortunei* (Hook.) H.Wendl. *	*Arecaceae*	Leaf	289803	Sevilla	134.0 ± 24.8	162.4 ± 13.6	1.5 ± 0.5
**80**	*Verbena officinalis* L.	*Verbenaceae*	Aerial part with flowers	289275	Sevilla	31.5 ± 21.7	4.2 ± 0.7	0.7 ± 0.4
**81**	*Xiphion xiphium* (L.) M.B. Crespo, Mart.-Azorín & Mavrodiev	*Iridaceae*	Flowers	289729	Huelva	8.6 ± 2.1	250.2 ± 29.5	38.9 ± 8.9
**82**	*Zelkova serrata* (Thunb.) Makino *	*Ulmaceae*	Aerial part	289263	Sevilla	173.4 ± 57.4	187.9 ± 43.0	1.6 ± 0.4
	Cisplatin					0.4 ± 0.1	1.0 ± 0.3	2.3 ± 1.1
	5FU					0.5 ± 0.1 (µM)	1.0 ± 0.5 (µM)	2.2 ± 1.5

Plants from cultures are marked with “*” after the scientific name. Voucher specimens were deposited in the Herbarium of the University of Seville (SEV). Selectivity index was calculated as the average of the IC_50_ value in the HaCaT non-malignant cell line divided by the IC_50_ value in the A549 cancer cell line obtained in each independent experiment; N.D.: not determined.

**Table 2:** IC_50_ values and selectivity indices of extract **81** and gemcitabine in melanoma (MeWo), bladder carcinoma (T24), and gastric cancer (KATO III) versus non-malignant cells (HaCaT).

Cell Line	Extract 81		Gemcitabine	
	IC_50_ (Mean ± SEM, µg/mL)	S.I. (Mean ± SEM)	IC_50_ (Mean ± SEM, nM)	S.I. (Mean ± SEM)
HaCaT (non-malignant keratinocytes)	321.3 ± 1.6	-	21.2 ± 8.1	-
MeWo (melanoma)	296.5 ± 19.1	1.1 ± 0.2	2.2 ± 0.9	17.5 ± 7.0
T24 (bladder cancer)	130.8 ± 13.5	2.6 ± 0.3	3.2 ± 0.2	11.0 ± 0.1
KATO III (gastric cancer)	305.4 ± 17.1	1.1 ± 0.1	1.4 ± 0.4	35.7 ± 12.0

S.I.: selectivity index (calculated as the average of the IC_50_ value in the HaCaT non-malignant cell line divided by the IC_50_ value in the cancer cell line obtained in each independent experiment).

## Data Availability

Data are contained within the article.

## Supplementary Materials

The following supporting information can be downloaded at: https://www.mdpi.com/article/10.3390/plants12183275/s1, Table S1. Collection coordinates of plants used in this work. Table S2. The extraction yield (%) for each extract used in this work. Figure S1. Evaluation of selective cytotoxic activity of plant extracts 1–7 and 9–12 on A549 lung cancer cells and HaCaT non-malignant cells. Figure S2. Evaluation of selective cytotoxic activity of plant extracts 14–25 on A549 lung cancer cells and HaCaT non-malignant cells. Figure S3. Evaluation of selective cytotoxic activity of plant extracts 26, 29–34, and 36–40 on A549 lung cancer cells and HaCaT non-malignant cells. Figure S4. Evaluation of selective cytotoxic activity of plant extracts 41, 42, 44, 45, and 48–55 on A549 lung cancer cells and HaCaT non-malignant cells. Figure S5. Evaluation of selective cytotoxic activity of plant extracts 56–62 and 64–68 on A549 lung cancer cells and HaCaT non-malignant cells. Figure S6. Evaluation of selective cytotoxic activity of plant extracts 69–80 on A549 lung cancer cells and HaCaT non-malignant cells. Figure S7. Evaluation of selective cytotoxic activity of plant extract 82 on A549 lung cancer cells and HaCaT non-malignant cells. Figure S8. Evaluation of selective cytotoxic activity of plant extract 81 on A549 lung cancer cells, HaCaT non-malignant cells, and BJ-hTERT non-malignant cells.
