# Supplementary material for: Evaluation of Anticancer Activity of 76 Plant Species Collected in Andalusia (Spain) against Lung Cancer Cells
Source: Plants (Basel). 2023 Sep 15;12(18):3275. doi: 10.3390/plants12183275 (PMC10536323; doi:10.3390/plants12183275)
Supplement: Supplementary file 1 [file plants-12-03275-s001.zip › plants-2566655-supplementary.pdf]

Article

Evaluation of Anticancer Activity of 76 Plant Species Collected in Andalusia (Spain) Against Lung Cancer Cells

Víctor Jiménez-González [1,*], Guillermo Benítez [2], Julio Enrique Pastor [3], Miguel López-Lázaro [1] and José Manuel Calderón-Montaño [1,*]

[1] Department of Pharmacology, Faculty of Pharmacy, University of Seville, 41012 Seville, Spain; mlopezlazaro@us.es
[2] Department of Botany, Faculty of Pharmacy, University of Granada, 18071 Granada, Spain; gbcruz@ugr.es
[3] Department of Vegetal Biology and Ecology, Faculty of Biology, University of Seville, 41012 Seville, Spain; jpastor@us.es
* Correspondence: vjimenez3@us.es (V.J.-G.); jcalderon@us.es (J.M.C.-M.)

Supplementary Materials

Table S1. Collection coordinates of plants used in this work.

Extract	Plant name	Coordinates
1	*Acoelorraphe wrightii* (Griseb. & H.Wendl.) H.Wendl. ex Becc.*	37º25'27'' N 5º59'42'' W
2	*Aegilops geniculata* Roth	37º43'59' N 6º10'13'' W
3	*Alkanna tinctoria* (L.) Tausch	37º20'25'' N 5º46'56'' W
4	*Alyssum simplex* Rudolphi	37º19'11'' N 5º46'20'' W
5	*Amaryllis belladonna* L.*	37º30'55'' N 6º23'28'' W
6	*Arisarum simorrhinum* Durieu	37º34'24'' N 6º 3' 38'' W
7	*Aristolochia paucinervis* Pomel	37º21'10'' N 5º47'50'' W
8	*Arum italicum* Mill. *subsp. italicum*	37º 34' 6'' N 6º 3' 14'' W
9	*Bartsia trixago* L.	37º41'13'' N 6º18'54'' W
10	*Bolboschoenus maritimus* (L.) Palla	37°19'15.5"N 6°02'25.1"W
11	*Brachychiton populneus* R.Br.*	37º25'22'' N 5º59'47'' W
12	*Briza maxima* L.	37º41'14'' N 6º18'19'' W
13	*Butia capitata* (Mart.) Becc.*	37º25'27'' N 5º59'41'' W
14	*Catalpa bignoniodes* Walter*	37º25'24'' N 5º59'34'' W
15	*Ceiba speciosa* (A.St.-Hil.) Ravenna*	37º25'22'' N 5º59'49'' W
16-17	*Celtis australis* L.	37º20'29'' N 5º46'43'' W
18	*Centranthus calcitrapae* (L.) Dufr.	37º47'0'' N 6º13'26'' W
19	*Cerinthe major* L.	37º22'33'' N 6º13'7'' W
20	*Ceterach officinarum* Willd. subsp. *officinarum*	37º34'17'' N 6º3'20'' W
21-22	*Chamaerops humilis* L.	37º16'55'' N 6º23'1'' W
23	*Cuscuta campestris* Yunck.	37°25'50.6"N 5°57'39.3"W
24	*Dipcadi serotinum* (L.) Medik.	37°16'21"N 6°23'52"W
25	*Fedia cornucopiae* (L.) Gaertn.	37º23'13'' N 6º13'30'' W

Extract	Plant name	Coordinates
26	*Firmiana simplex* (L.) W.Wight*	37º25'24'' N 5º59'36'' W
27-28	*Gynandriris sisyrinchium* (L.) Parl.	37°19'49"N 5°47'37"W
29	*Heliotropium europaeum* L.	37º19'7'' N 5º46'2'' W
30	*Iris germanica* L.	37º30'55'' N 6º23'28'' W
31	*Jacaranda mimosifolia* D.Don*	37°24'54.8"N 6°00'18.7"W
32-33	*Jasminum fruticans* L.	37º17'29'' N 6º7'39'' W
34	*Juncus acutus* L. *subsp. acutus*	37º22'27'' N 5º44'51'' W
35	*Juno planifolia* (Mill.) Asch.	37°23'45.6"N 6°13'46.2"W
36	*Koelreuteria paniculata* Laxm.*	37º25'25'' N 5º59'43'' W
37	*Lagerstroemia indica* L.*	37º25'22'' N 5º 59' 38'' W
38	*Lagerstroemia speciosa* (L.) Pers.*	37º25'24'' N 5º59'38'' W
39	*Lagunaria patersonia* (Andrews) G. Don*	37º25'24'' N 5º59'36'' W
40	*Linaria viscosa (L.) Chaz.*	37º22'16'' N 6º13'21'' W
41	*Liquidambar styraciflua* L.*	37º25'24'' N 5º59'34'' W
42	*Lolium rigidum* Gaudin	37º18'21'' N 6º2'32'' W
43	*Lomelosia simplex* (Desf.) Raf. subsp. *dentata* (Jord. & Fourr.) Greuter & Burde	37º16'34'' N 6º23'26'' W
44	*Lonicera implexa* Aiton	37º30'12'' N 6º23'41'' W
45	*Maclura pomifera* (Raf.) C.K.Schneid.*	37º25'20'' N 5º59'39'' W
46-47	*Mandragora autumnalis* Bertol.	37º22'40''N 6º10'30'' W
48	*Morus nigra* L.	37°19'04.6"N 6°02'48.4"W
49	*Muscari comosum* (L.) Mill.	37º39'28'' N 6º13'31'' W
50	*Nonea vesicaria* (L.) Rchb.	37º22'45'' N 6º13'33'' W
51	*Oenothera rosea* L'Hér. ex Aiton*	37º25'21'' N 5º59'40'' W
52	*Ophrys scolopax* Cav.	37º21'10'' N 5º47'50'' W
53	*Ophrys speculum* Link	37º20'16'' N 5º47'14'' W
54	*Ornithogalum baeticum* Boiss.*	37º21'10'' N 5º47'50'' W
55	*Orobanche crenata* Forssk.	37º38'59'' N 6º12'27'' W
56	*Parentucellia viscosa* (L.) Caruel	37°19'00.3"N 6°03'00.4"W
57	*Paronychia argentea* Lam.	37º29'28'' N 6º13'31'' W
58	*Petrorhagia nanteuilii* (Burnat) P.W.Ball & Heywood	37º41'11'' N 6º18'58'' W
59	*Photinia glabra* (Thunb.) Poit..*	37º25'24'' N 5º59''33'' W
60	*Platanus hispanica* Mill. ex Münchh.*	37º25'21'' N 5º59'43'' W
61	*Platycapnos spicata* (L.) Bernh.	37º21'45'' N 6º 12'22'' W
62	*Plumbago europaea* L.	37º19'7'' N 5º46'2'' W
63-64	*Rhamnus alaternus* L.	37º41'28'' N 6º18'47'' W
65	*Rosa canina* L.	37º22'31''N 6º13'29'' W
66	*Rumex conglomeratus* Murray	37º15'9'' N 6º22'33'' W
67	*Schinus molle* L.	37º25'23'' N 5º59'40'' W
68	*Scirpoides holoschoenus* (L.) Soják	37º13'56'' N 5º42'59'' W
69	*Scrophularia sambucifolia* L.	37º18'16'' N 5º43'44'' W
70	*Sedum amplexicaule* DC. subsp. *amplexicaule*	37º43'59'' N 6º10'13'' W
71	*Sedum mucizonia* (Ortega) Raym.-Hamet	37º41'16'' N 6º18'58'' W
72	*Solandra maxima* (Moc. & Sessé ex Dunal) P.S.Green*	37º23'52'' N 6º 0'17'' W
73	*Solanum nigrum* L.	37º34'6'' N 6º3'14'' W

Extract	Plant name	Coordinates
74	*Swietenia mahagoni* (L.) Jacq.*	37º25'27'' N 5º59'41'' W
75	*Syagrus romanzoffiana* (Cham.) Glassman*	37º 25'20'' N 5º59'41'' W
76	*Taxodium distichum* (L.) Rich.*	37º 25'28'' N 5º59'40'' W
77	*Thymbra capitata* (L.) Cav.	37º20'56'' N 5º47'59'' W
78	*Tilia tomentosa* Moench.*	37º25'19'' N 5º 59'35'' W
79	*Trachycarpus fortunei* (Hook.) H.Wendl.*	37º25'21'' N 5º59'40'' W
80	*Verbena officinalis* L.	37º21' 10'' N 5º45'8'' W
81	*Xiphion xiphium* (L.) M.B. Crespo, Mart.-Azorín & Mavrodiev	37º17'9'' N 6º23'21'' W
82	*Zelkova serrata* (Thunb.) Makino*	37º25'20'' N 5º59'33'' W

Plants from cultures are marked with "*" after the scientific name.

Table S2. The extraction yield (%) for each extract used in this work.

Extract	Plant name	Part used	Extraction yield (%)
1	*Acoelorraphe wrightii* (Griseb. & H.Wendl.) H.Wendl. ex Becc.*	Leaf	6.9
2	*Aegilops geniculata* Roth	Whole plant	5.8
3	*Alkanna tinctoria* (L.) Tausch	Aerial part with flowers	3.9
4	*Alyssum simplex* Rudolphi	Whole plant	3.6
5	*Amaryllis belladonna* L.*	Root	0.4
6	*Arisarum simorrhinum* Durieu	Aerial parts with flowers	2.0
7	*Aristolochia paucinervis* Pomel	Aerial part with flowers	5.6
8	*Arum italicum* Mill. *subsp. italicum*	Aerial parts	19.6
9	*Bartsia trixago* L.	Aerial part with flowers	8.5
10	*Bolboschoenus maritimus* (L.) Palla	Aerial part with flowers	3.5
11	*Brachychiton populneus* R.Br.*	Aerial parts with fruits	3.5
12	*Briza maxima* L.	Aerial part	3.8
13	*Butia capitata* (Mart.) Becc.*	Leaf	1.8
14	*Catalpa bignoniodes* Walter*	Leaf	8.9
15	*Ceiba speciosa* (A.St.-Hil.) Ravenna*	Aerial part	4.3
16	*Celtis australis* L.	Fruits	8.0
17	*Celtis australis* L.	Aerial part	4.4

Extract	Plant name	Part used	Extraction yield (%)
18	*Centranthus calcitrapae* (L.) Dufr.	Aerial part with flowers	7.2
19	*Cerinthe major* L.	Aerial parts with flowers	5.0
20	*Ceterach officinarum* Willd. subsp. *officinarum*	Aerial parts	7.5
21	*Chamaerops humilis* L.	Leaves	5.6
22	*Chamaerops humilis* L.	Fruits	6.0
23	*Cuscuta campestris* Yunck.	Aerial part with flowers	4.3
24	*Dipcadi serotinum* (L.) Medik.	Whole plant	3.2
25	*Fedia cornucopiae* (L.) Gaertn.	Whole plant	3.6
26	*Firmiana simplex* (L.) W.Wight*	Leaf	6.6
27	*Gynandriris sisyrinchium* (L.) Parl.	Whole plant	3.3
28	*Gynandriris sisyrinchium* (L.) Parl.	Flowers	2.5
29	*Heliotropium europaeum* L.	Aerial part with flowers	1.4
30	*Iris germanica* L.	Root	3.2
31	*Jacaranda mimosifolia* D.Don*	Flowers	8.9
32	*Jasminum fruticans* L.	Fruits	4.6
33	*Jasminum fruticans* L.	Aerial parts	10.9
34	*Juncus acutus* L. *subsp. acutus*	Aerial part with fruits	1.7
35	*Juno planifolia* (Mill.) Asch.	Aerial parts with flowers	3.4
36	*Koelreuteria paniculata* Laxm.*	Leaf	5.5
37	*Lagerstroemia indica* L.*	Aerial part	4.1
38	*Lagerstroemia speciosa* (L.) Pers.*	Leaf	4.5
39	*Lagunaria patersonia* (Andrews) G. Don*	Leaf	3.4
40	*Linaria viscosa (L.) Chaz.*	Aerial parts with flowers	6.2
41	*Liquidambar styraciflua* L.*	Aerial part	6.7
42	*Lolium rigidum* Gaudin	Aerial parts	4.5
43	*Lomelosia simplex* (Desf.) Raf. subsp. *dentata* (Jord. & Fourr.) Greuter & Burde	Aerial part with flowers	8.4
44	*Lonicera implexa* Aiton	Leaves	10.2
45	*Maclura pomifera* (Raf.) C.K.Schneid.*	Aerial part	7.2
46	*Mandragora autumnalis* Bertol.	Flower and fruits	2.5
47	*Mandragora autumnalis* Bertol.	Whole plant	2.8
48	*Morus nigra* L.	Fruits	4.2
49	*Muscari comosum* (L.) Mill.	Aerial parts with flowers	2.7
50	*Nonea vesicaria* (L.) Rchb.	Whole plant	5.6
51	*Oenothera rosea* L'Hér. ex Aiton*	Aerial parts with flowers	5.2
52	*Ophrys scolopax* Cav.	Aerial part with flowers	7.5
53	*Ophrys speculum* Link	Whole plant	2.8
54	*Ornithogalum baeticum* Boiss.*	Whole plant	1.3
55	*Orobanche crenata* Forssk.	Aerial parts with flowers	4.1
56	*Parentucellia viscosa* (L.) Caruel	Aerial part with flowers	3.3

Extract	Plant name	Part used	Extraction yield (%)
57	*Paronychia argentea* Lam.	Whole plant	4.7
58	*Petrorhagia nanteuilii* (Burnat) P.W.Ball & Heywood	Aerial part with flowers	2.7
59	*Photinia glabra* (Thunb.) Poit..*	Aerial part	9.8
60	*Platanus hispanica* Mill. ex Münchh.*	Leaf	5.7
61	*Platycapnos spicata* (L.) Bernh.	Aerial parts with flowers	6.3
62	*Plumbago europaea* L.	Aerial part with flowers and fruits	3.9
63	*Rhamnus alaternus* L.	Leaves	5.9
64	*Rhamnus alaternus* L.	Fruits	3.8
65	*Rosa canina* L.	Fruits	6.7
66	*Rumex conglomeratus* Murray	Aerial part with flowers	5.2
67	*Schinus molle* L.	Aerial part	6.2
68	*Scirpoides holoschoenus* (L.) Soják	Aerial part with fruits	1.8
69	*Scrophularia sambucifolia* L.	Aerial part with flowers	5.0
70	*Sedum amplexicaule* DC. subsp. *amplexicaule*	Whole plant	2.3
71	*Sedum mucizonia* (Ortega) Raym.-Hamet	Whole plant	2.8
72	*Solandra maxima* (Moc. & Sessé ex Dunal) P.S.Green*	Leaves	2.9
73	*Solanum nigrum* L.	Aerial parts	31.9
74	*Swietenia mahagoni* (L.) Jacq.*	Leaf	6.3
75	*Syagrus romanzoffiana* (Cham.) Glassman*	Aerial part	6.1
76	*Taxodium distichum* (L.) Rich.*	Aerial part	8.4
77	*Thymbra capitata* (L.) Cav.	Aerial part with flowers	0.7
78	*Tilia tomentosa* Moench.*	Leaf	2.5
79	*Trachycarpus fortunei* (Hook.) H.Wendl.*	Leaf	6.5
80	*Verbena officinalis* L.	Aerial part with flowers	3.7
81	*Xiphion xiphium* (L.) M.B. Crespo, Mart.-Azorín & Mavrodiev	Flowers	1.4
82	*Zelkova serrata* (Thunb.) Makino*	Aerial part	1.9

Plants from cultures are marked with "*" after the scientific name.

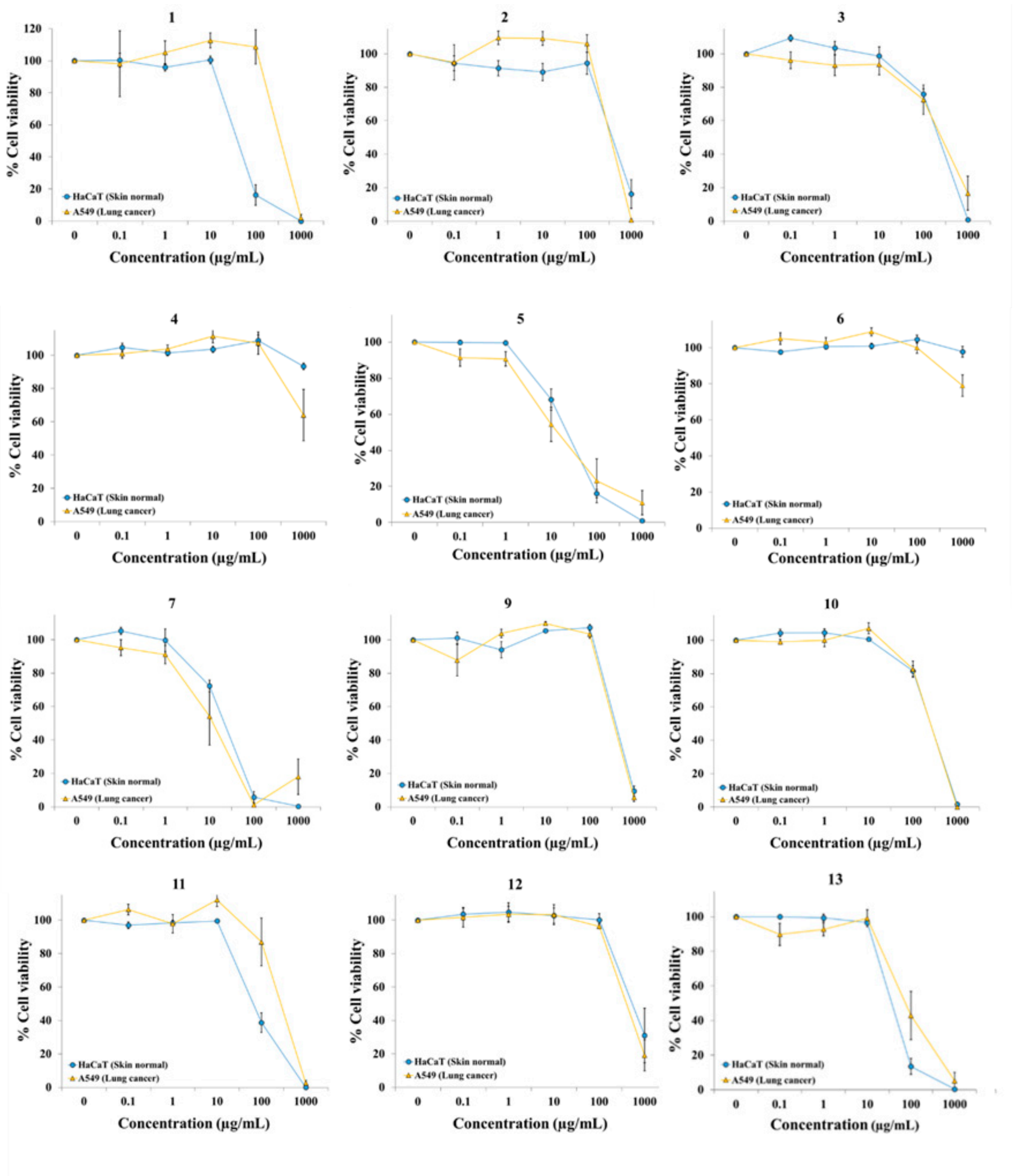

Figure S1. Evaluation of selective cytotoxic activity of plant extracts 1–7, 9-13 on A549 lung cancer cells and HaCaT non-malignant cells. The cells were exposed for 72 h to the extracts and cell viability was determined with the resazurin assay.

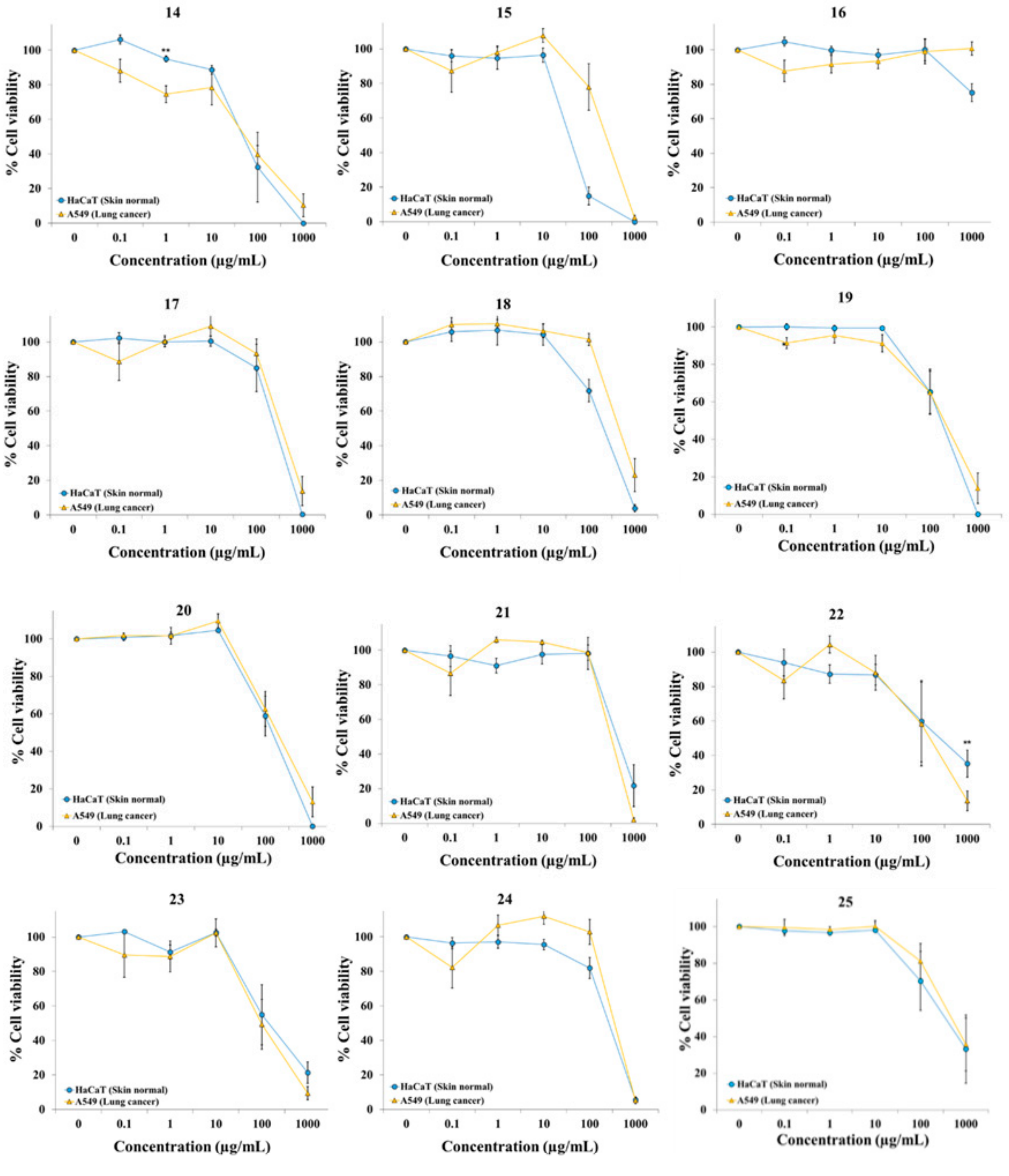

Figure S2. Evaluation of selective cytotoxic activity of plant extracts 14-25 on A549 lung cancer cells and HaCaT non-malignant cells. The cells were exposed for 72 h to the extracts and cell viability was determined with the resazurin assay.

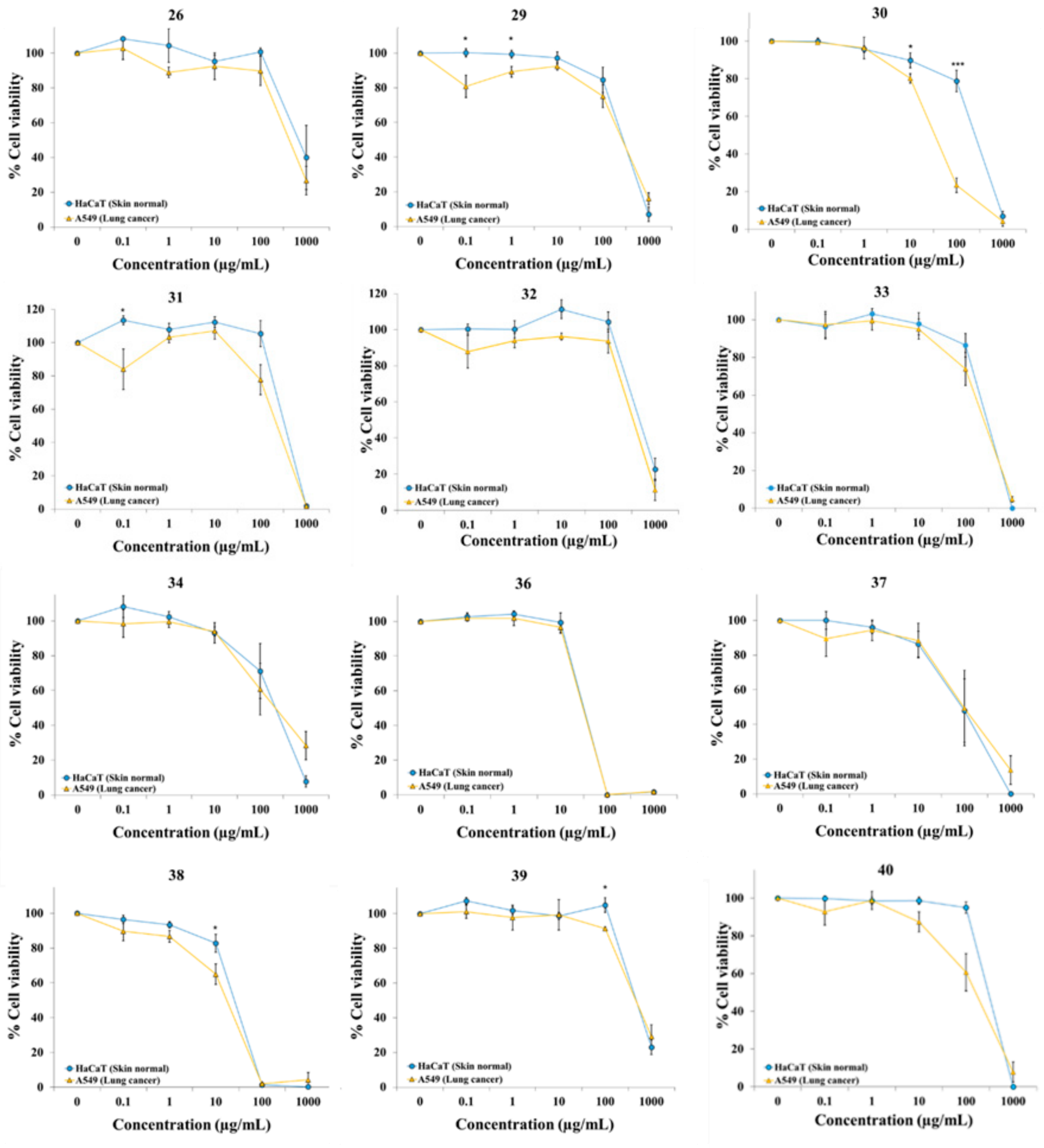

Figure S3. Evaluation of selective cytotoxic activity of plant extracts 26, 29-34, 36-40 on A549 lung cancer cells and HaCaT non-malignant cells. The cells were exposed for 72 h to the extracts and cell viability was determined with the resazurin assay.

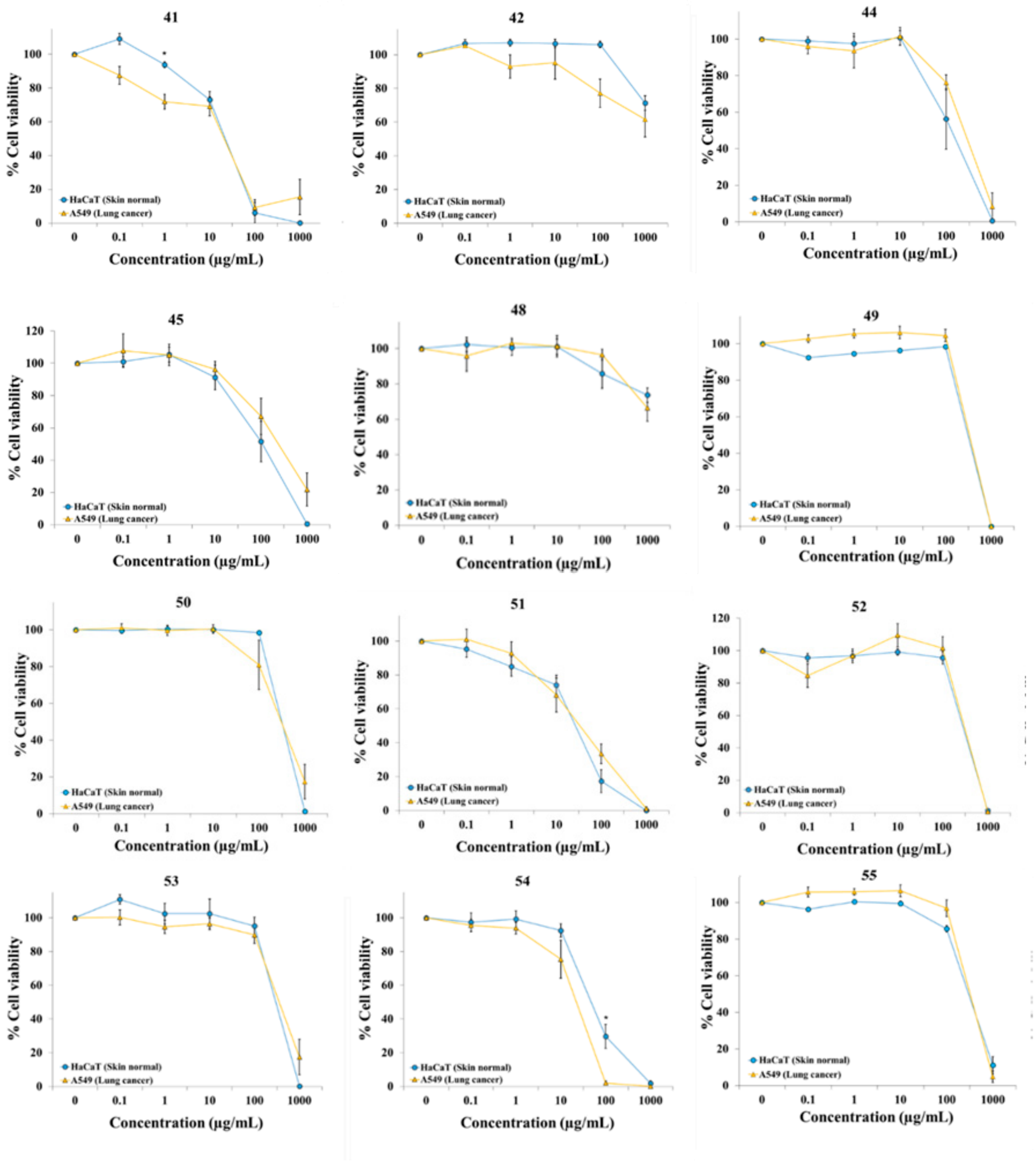

Figure S4. Evaluation of selective cytotoxic activity of plant extracts 41, 42, 44, 45, 48-55 on A549 lung cancer cells and HaCaT non-malignant cells. The cells were exposed for 72 h to the extracts and cell viability was determined with the resazurin assay.

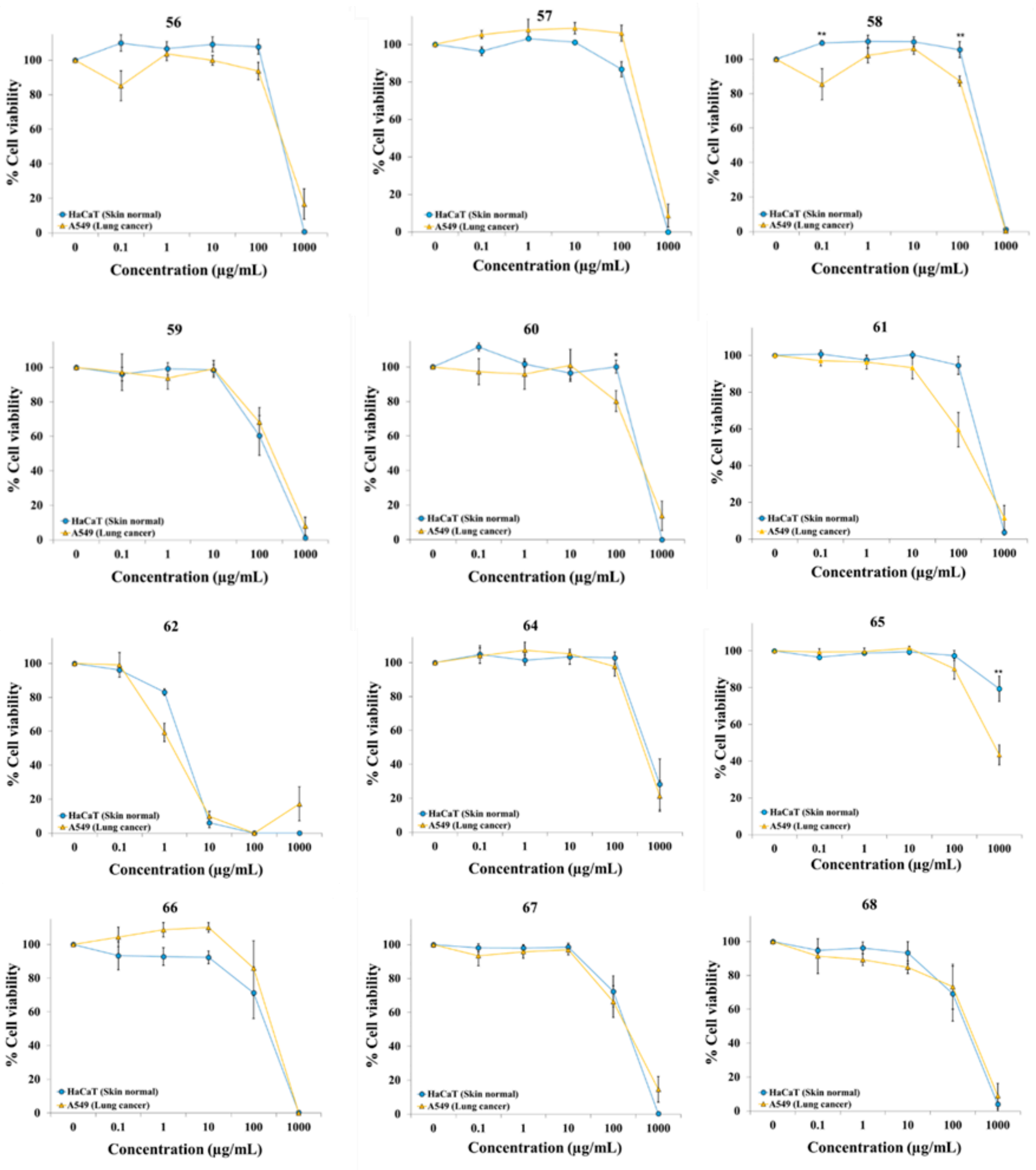

Figure S5. Evaluation of selective cytotoxic activity of plant extracts 56-62, 64-68 on A549 lung cancer cells and HaCaT non-malignant cells. The cells were exposed for 72 h to the extracts and cell viability was determined with the resazurin assay.

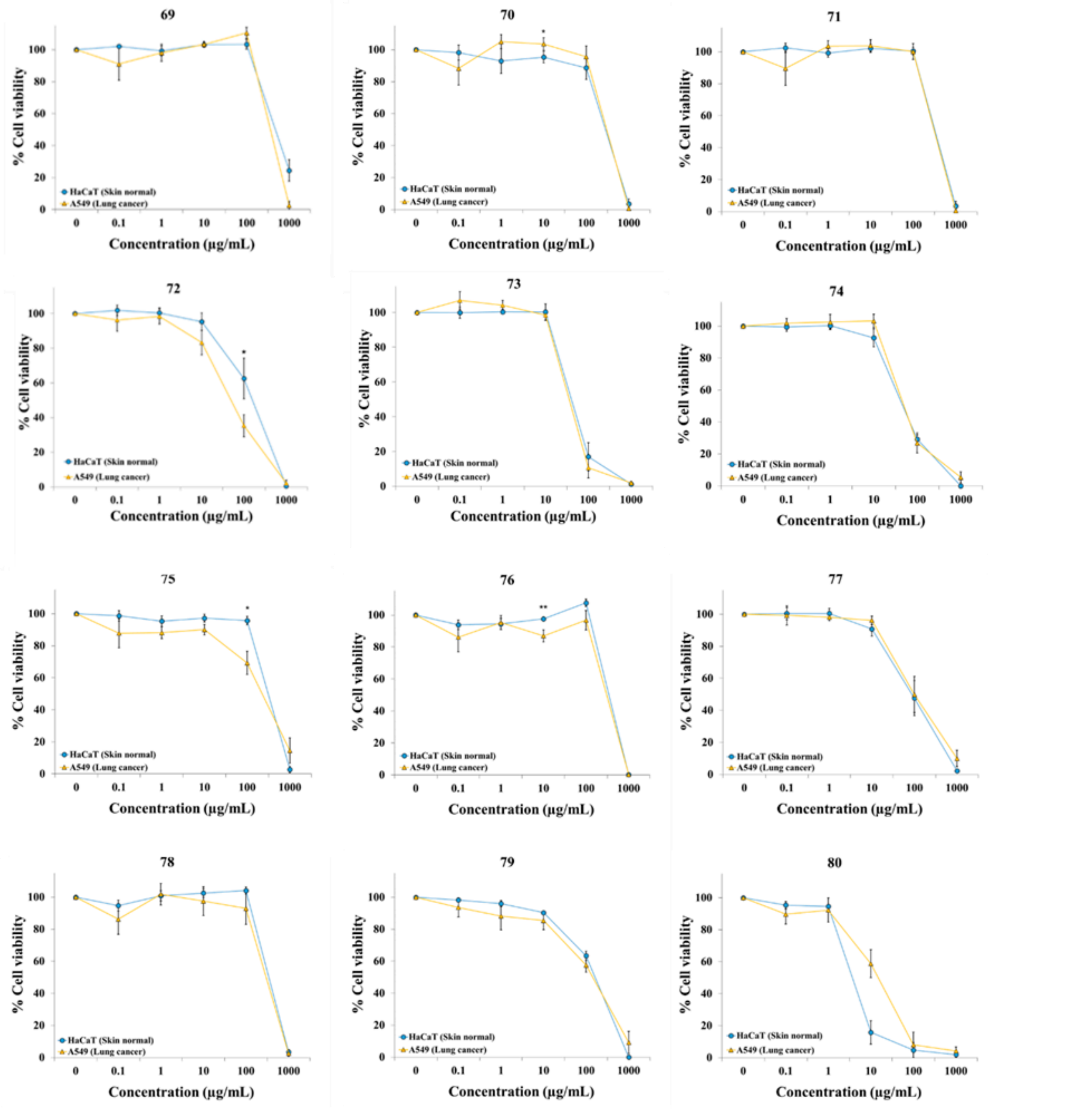

Figure S6. Evaluation of selective cytotoxic activity of plant extracts 69-80 on A549 lung cancer cells and HaCaT non-malignant cells. The cells were exposed for 72 h to the extracts and cell viability was determined with the resazurin assay.

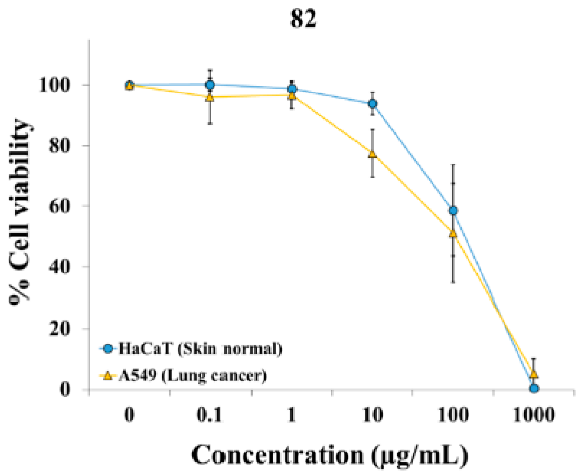

Figure S7. Evaluation of selective cytotoxic activity of plant extract 82 on A549 lung cancer cells and HaCaT non-malignant cells. The cells were exposed for 72 h to the extracts and cell viability was determined with the resazurin assay.

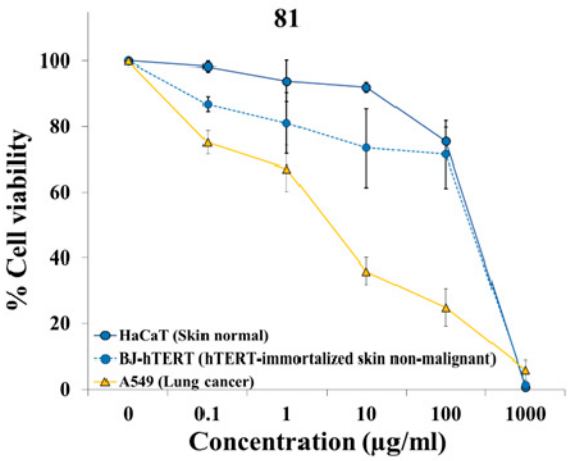

Figure S8. Evaluation of selective cytotoxic activity of plant extract 81 on A549 lung cancer cells, HaCaT non-malignant cells and BJ-hTERT non-malignant cells. The cells were exposed for 72 h to the extracts and cell viability was determined with the resazurin assay. Data represent mean ± SEM from at least two independent experiments.
